# The association of appendicular lean mass and grip strength with low-density lipoprotein, very low-density lipoprotein, and high-density lipoprotein particle diameter: a Mendelian randomization study of the UK Biobank cohort

**DOI:** 10.1093/ehjopen/oeae019

**Published:** 2024-03-14

**Authors:** Richard Kirwan, Mohsen Mazidi, Tom Butler, Fatima Perez de Heredia, Gregory Y H Lip, Ian G Davies

**Affiliations:** Research Institute of Sport and Exercise Science, Liverpool John Moores University, Liverpool, UK; Liverpool Centre for Cardiovascular Science at University of Liverpool, Liverpool John Moores University, Liverpool Heart and Chest Hospital, Liverpool, UK; Liverpool Centre for Cardiovascular Science at University of Liverpool, Liverpool John Moores University, Liverpool Heart and Chest Hospital, Liverpool, UK; Clinical Trial Service Unit, Epidemiological Studies Unit, Nuffield Department of Population Health, University of Oxford, Old Road Campus, Roosevelt Dr., Doll Bldg, Oxford, OX3 7LF, UK; Department of Twin Research and Genetic Epidemiology, King's College London, London, UK; School of Applied Health and Social Care and Social Work, Faculty of Health, Social Care and Medicine, Edge Hill University, Ormskirk, UK; School of Biological and Environmental Sciences, Liverpool John Moores University, Liverpool, UK; Liverpool Centre for Cardiovascular Science at University of Liverpool, Liverpool John Moores University, Liverpool Heart and Chest Hospital, Liverpool, UK; Danish Center for Clinical Health Services Research, Department of Clinical Medicine, Aalborg University, Aalborg, Denmark; Research Institute of Sport and Exercise Science, Liverpool John Moores University, Liverpool, UK; Liverpool Centre for Cardiovascular Science at University of Liverpool, Liverpool John Moores University, Liverpool Heart and Chest Hospital, Liverpool, UK

**Keywords:** Muscle mass, Mendelian randomization, Low-density lipoprotein, Sarcopenia, Lean mass, Lipoprotein diameter, High-density lipoprotein

## Abstract

**Aims:**

Reduced muscle mass and reduced strength are frequently associated with both alterations in blood lipids and poorer cardiometabolic outcomes in epidemiological studies; however, a causal association cannot be determined from such observations. Two-sample Mendelian randomization (MR) was applied to assess the association of genetically determined appendicular lean mass (ALM) and handgrip strength (HGS) with serum lipid particle diameter.

**Methods and results:**

Mendelian randomization was implemented using summary-level data from the largest genome-wide association studies on ALM (*n* = 450 243), HGS (*n* = 223 315), and lipoprotein [low-density lipoprotein (LDL), very LDL (VLDL), and high-density lipoprotein (HDL)] particle diameters (*n* = 115 078). Inverse variance-weighted (IVW) method was used to calculate the causal estimates. Weighted median-based method, MR-Egger, and leave-one-out method were applied as sensitivity analysis. Greater ALM had a statistically significant positive effect on HDL particle diameter (MR-Egger: *β* = 0.055, SE = 0.031, *P* = 0.081; IVW: *β* = 0.068, SE = 0.014, *P* < 0.001) and a statistically significant negative effect on VLDL particle diameter (MR-Egger: *β* = −0.114, SE = 0.039, *P* = 0.003; IVW: *β* = −0.081, SE = 0.017, *P* < 0.001). Similarly, greater HGS had a statistically significant positive effect on HDL particle diameter (MR-Egger: *β* = 0.433, SE = 0.184, *P* = 0.019; IVW: *β* = 0.121, SE = 0.052, *P* = 0.021) and a statistically significant negative effect on VLDL particle diameter (MR-Egger: *β* = −0.416, SE = 0.163, *P* = 0.011; IVW: *β* = −0.122, SE = 0.046, *P* = 0.009). There was no statistically significant effect of either ALM or HGS on LDL particle diameter.

**Conclusion:**

There were potentially causal associations between both increasing ALM and HGS and increasing HDL particle size and decreasing VLDL particle size. These causal associations may offer possibilities for interventions aimed at improving cardiovascular disease risk profile.

## Introduction

Cardiovascular diseases (CVDs) are the leading cause of mortality globally, resulting in 18.6 million deaths in 2019 alone.^1^ Reduced muscle mass and reduced grip strength have both been associated with increased risk of CVD^2,3^ and CVD mortality.^4,5^ Muscle mass is known to decline progressively from the fifth decade of life, a process known as sarcopenia,^6^ a condition that is also associated with CVD.^7^ Greater muscle mass and function are associated with favourable levels of a number of relevant risk factors for CVD, including plasma triglycerides^8^ and high-density lipoprotein cholesterol (HDL-C),^9,10^ although some unfavourable associations have also been observed.^11^ However, the relationships between muscle mass/strength and low-density lipoprotein (LDL), very LDL (VLDL), and HDL particle diameter remain to be investigated.

Elevated LDL levels are causally associated with the risk of CVD and, particularly, coronary heart disease (CHD).^12^ Indeed, long-term cumulative exposure to elevated LDL-cholesterol (LDL-C) from a young age (for example due to genetic predisposition to higher LDL-C) is associated with a greater risk of incident CVD events.^13,14^ Furthermore, LDL and other lipoprotein particle sizes can be measured using techniques such as nuclear magnetic resonance (NMR) spectroscopy and sub-fractionation. Accordingly, LDL is known to have particle subclasses, commonly divided into small, medium, and large, according to their diameter.^15^ Small dense LDL (sdLDL) particles, despite their lower cholesterol load, may contribute equally to larger LDL to CVD risk due to their greater propensity to enter and become trapped in the sub-intimal space of the arterial wall, contributing to the development of atherosclerosis.^16–18^ Very LDL is another subclass of lipoprotein that is considered to be atherogenic, with larger VLDL particles linked to a greater risk of the development of CVD in healthy populations.^19,20^ Conversely, lower levels of HDL-C are associated with increased CVD risk,^21^ while smaller HDL particle size is associated with an adverse cardiometabolic risk profile.^20,22^ Indeed, in the largest study to date of major lipoprotein subclasses, CHD risk was most strongly related to VLDL, LDL, and HDL particle concentrations.^23^ However, the relationship between muscle mass and the size of these serum lipid particles is unknown.

Genome-wide association studies (GWAS) test great numbers of genes across multiple genomes to discover genetic variants statistically associated with a specific trait, such as muscle mass or strength.^24^ Such studies have revealed that genetic variants explain approximately 15.5% of the phenotypic variance in appendicular lean mass (ALM).^25^ Similar genetic associations are observed for handgrip strength (HGS), which is related to genetic variations in the structure and function of skeletal muscle fibres and neuronal transduction in the central and peripheral nervous systems.^26^ However, despite epidemiological associations of lower muscle mass and strength with poorer cardiometabolic risk markers and outcomes,^2–5^ causality cannot be determined from such observations. In contrast, Mendelian randomization (MR) analysis uses genetic polymorphisms known to be associated with distinct alterations in phenotypes (for example, genetically determined ALM), as statistical instruments.^27^ This allows the determination of whether a particular physiological trait is a probable cause of a known risk factor or specific condition.^27^ This means MR analysis is capable of determining both unbiased and robust evidence of the mechanisms of disease pathogenesis. A further advantage of MR analysis is that it is considerably less prone to confounding, residual bias, and reverse causation than conventional risk factor epidemiology.^28^ As such, data from MR analysis can inform the design of pilot randomised controlled trials (RCTs) and clinical trials by identifying potential treatment targets and even the magnitude of the effect of targeted treatments in specific populations.^29^

In the present study, we used MR analysis to determine the relationship between genetically determined ALM and HGS with lipid indices associated with an adverse cardiometabolic risk profile, i.e. LDL, VLDL, and HDL particle sizes.

## Methods

### Study design

In this MR investigation, we utilized a two-sample approach, sourcing aggregate data from multiple studies to examine the correlation between genetic instruments with both the exposures and the outcomes. The data for muscle mass (ALM) (*n* = 450 243)^25^ and HGS (*n* = 223 315),^30^ and the exposures, along with lipoprotein particle sizes, and the outcomes (*n* = 115 078) were extracted from the most comprehensive GWAS available. To discern the causal influence of ALM and HGS on the dimensions of LDL, HDL, and VLDL particles, we implemented analytical strategies designed to yield unbiased effects.

### Genetic predictors of exposures

We used single nucleotide polymorphisms (SNPs) identified to be associated with ALM from the UK Biobank,^25^ with samples of self-reported white ancestry (*n* = 450 243) and partial replication in a smaller population of South-Asian ancestry (*n* = 7452). The UK Biobank is a population-based cohort of approximately 500 000 individuals; 54% are female, the average age is 57 years (range 37–73), and 94% report being White British. Further details on the rationale, design, and methodology for UK Biobank can be found elsewhere.^31^ Comprehensive methodologies detailing the assessment of body composition can be found on the UK Biobank’s resource center.^32^ In summary, bioelectrical impedance analysis (BIA), utilizing the Tanita BC418MA body composition analyser, was employed to gauge both whole body and regional (trunk, leg, and arm) fat-free mass (FFM), alongside fat mass. Additionally, dual-energy X-ray absorptiometry was used to measure body composition in a participant sub-group, revealing a strong concordance with bio-impedance measurements for FFM (*r* = 0.96).^32^

We used SNPs identified to be associated with HGS also from the UK Biobank,^30^ with self-reported White British or European Caucasian ancestry (*n* = 223 315). Briefly, HGS was measured using a Jamar J00105 hydraulic hand dynamometer. Full methodology is described elsewhere.^33^ Uniform analysis protocols were adhered to during the execution of GWAS across each participant cohort. We employed additive genetic models, applying linear regression to the natural-log-transformed ALM or HGS, and subsequently, an inverse variance-weighted (IVW) meta-analysis with fixed effects was conducted to synthesize data from all contributing cohorts.^25,30^

### Association of genetic instruments with outcome

The associations of genetic instruments with SNPs associated with NMR-determined lipoprotein particle size were retrieved using data obtained from the Medical Research Council Integrative Epidemiology Unit Open GWAS project.^34,35^ Data were derived from a population of 115 078 men and women of European descent.

### Mendelian randomization analysis

Genetic instrument effects were combined using the IVW method as delineated in the two-sample MR package within the R statistical software program (R Core Team, Vienna, Austria. https://www.R-project.org/). Heterogeneity in effects was assessed via the *Q* statistic for IVW. To counter the potential influence of pleiotropic variants on our estimated effects, we executed a sensitivity analysis that included the weighted median (WM) and MR-Egger methods. The leave-one-out method was employed for sensitivity analysis. Provided that SNPs accounting for ≥50% of the weight are valid instruments, the WM estimate, representing the median of the SNP-specific-estimates weighted by their variance, will yield accurate estimates. This method utilizes IVW and bootstrapping techniques to determine confidence intervals.^36^ Mendelian randomization-Egger is capable of providing estimates under the premise that all SNPs are invalid instruments, provided that the instrument strength independent of direct effect assumption holds true.^36^

While MR-Egger permits the estimation of the intercept to be unrestricted, the confirmation of additional assumptions such as the independence between the instrument’s strength and its direct effects cannot be easily verified. The extent of average directional pleiotropy across the genetic variants was assessed using the *P*-value for the MR-Egger intercept term.^36^ It is noted that causal estimations derived from MR-Egger tend to be less precise compared with those obtained through IVW MR.^37^ Furthermore, MR-Egger analysis presents a lower rate of false positives but a higher rate of false negatives in comparison with the IVW method.^38^

To evaluate the heterogeneity across individual genetic variant estimates, the *Q*′ heterogeneity statistic^39^ and the MR pleiotropy residual sum and outlier (MR-PRESSO) test^39^ were utilized. The *Q*′ statistic employs modified second-order weights derived from a Taylor series expansion, which considers the uncertainty present in both the numerator and the denominator of the instrumental variable ratio, thereby relaxing the no-measurement-error assumption.^39^ The MR-PRESSO method is based on the regression analysis of variant-outcome associations against variant–exposure associations and employs a global heterogeneity test. This test juxtaposes the actual distance (residual sum of squares) of all variants and the regression line with the expected distance under the null hypothesis that no pleiotropy exists.^40^ If horizontal pleiotropy is detected, the test scrutinizes the distributions of individual variants, comparing expected and actual values to pinpoint outliers. Additionally, we employed the MR-Robust Adjusted Profile Score (RAPS), which is adept at adjusting for pleiotropy through RAPS. The results we considered for causality are those estimates that are consistent in direction and magnitude across various MR methods, achieve nominal significance via IVW MR, and show no indication of horizontal pleiotropy bias as per heterogeneity tests. These analyses were performed using R version 3.4.2 (R Core Development Team).

Mendelian randomization analyses rest on the premise that selected SNPs, serving as instrumental variables, are linked to the outcome solely through their impact on the exposure.^41^ Accordingly, we conducted a sensitivity analysis that omitted SNPs suspected of having pleiotropic effects. The instrumental variable analysis was evaluated against the ‘exclusion-restriction’ assumption using the Ensembl database (http://useast.ensembl.org/index.html), which provides a comprehensive catalogue of SNP phenotypes.

### Ethics

Our study is based on the analysis of previously published or publicly accessible summary data sets; thus, there was no direct engagement with study participants. No novel data were gathered specifically for this paper. Ethical clearances for the studies utilized in this analysis, as well as the informed consent obtained from every participant, are documented within the respective original publications.

## Results

**Figure 1:**
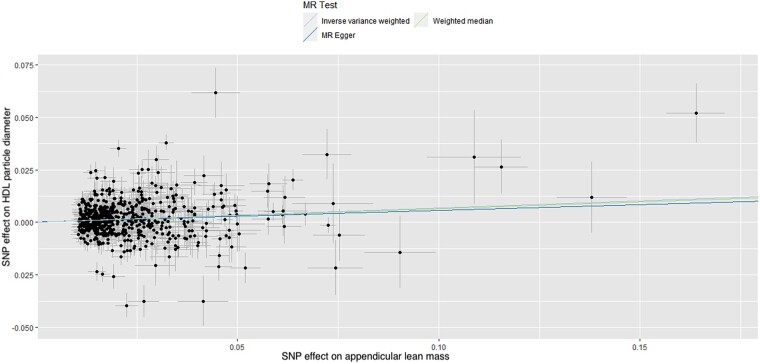
Scatter plot of the association of the effect of single nucleotide polymorphism-determined serum appendicular lean mass on high-density lipoprotein particle diameter. Each point represents a single nucleotide polymorphism, plotted by the estimate of single nucleotide polymorphism on appendicular lean mass (*x*-axis, kg) and the estimate of single nucleotide polymorphism on high-density lipoprotein particle diameter (*y*-axis, nm). The slopes of each line represent the potential causal associations for each method. HDL, high-density lipoprotein; MR, high-density lipoprotein; SNP, single nucleotide polymorphism.

**Figure 2:**
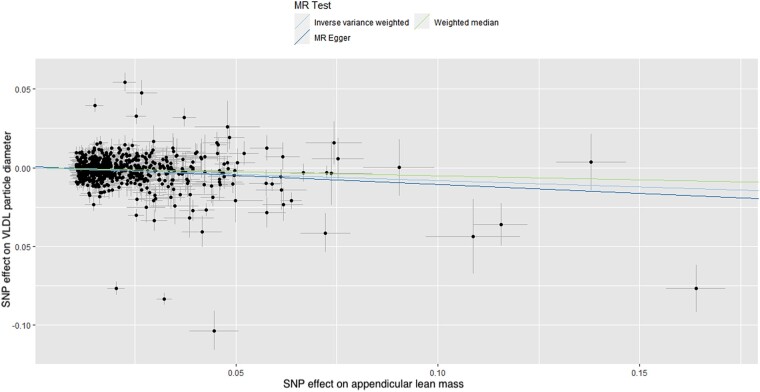
Scatter plot of the association of the effect of single nucleotide polymorphism-determined serum appendicular lean mass on very low-density lipoprotein particle diameter. Each point represents an single nucleotide polymorphism, plotted by the estimate of single nucleotide polymorphism on appendicular lean mass (*x*-axis, kg) and the estimate of single nucleotide polymorphism on very low-density lipoprotein particle diameter (*y*-axis, nm). The slopes of each line represent the potential causal associations for each method. VLDL, very low-density lipoprotein; MR, high-density lipoprotein; SNP, single nucleotide polymorphism.

In total, 608 and 169 SNPs were identified as instrumental variables for ALM and right HGS, respectively, none of which were significantly associated with LDL, VLDL, or HDL particle diameter, indicating a low risk of SNPs affecting multiple phenotypes via independent biological pathways. The results of MR analysis, displayed as beta-coefficient for interested outcomes per unit increase in ALM, demonstrated a statistically significant positive effect on HDL particle diameter (MR-Egger: *β* = 0.055, SE = 0.031, *P* = 0.081, and IVW: *β* = 0.068, SE = 0.014, *P* < 0.001, respectively; *Table 1* and *Figure 1*) and a statistically significant negative effect on VLDL particle diameter (MR-Egger: *β* = −0.114, SE = 0.039, *P* = 0.003, and IVW: *β* = −0.081, SE = 0.017, *P* < 0.001, respectively; *Table 1* and *Figure 2*).

**Table 1:** Results of the Mendelian randomization analysis for effects of genetically determined appendicular lean mass and handgrip strength on low-density lipoprotein, very low-density lipoprotein, and high-density lipoprotein particle size

Exposure	Outcome	MR				Heterogeneity			Pleiotropy		
		Method	beta	SE	*P*-value	Method	*Q*	*P*-value	Intercept	SE	*P*-value
**Appendicular lean mass**	LDL	MR Egger	0.03541	0.02624	0.1777	MR-Egger	987.3	9.8 × 10 ^−23^	−0.001	0.0006	0.078
		WM	−0.002108	0.0154	0.8911						
		IVW	−0.006395	0.01139	0.5745	IVW	992.6	4.3 × 10 ^−23^			
		RAPS	−0.02011	0.03798	0.5967						
	VLDL	MR Egger	−0.1137	0.0387	0.003427	MR-Egger	2359.7	3.9 × 10^−210^	0.0008	0.0009	0.341
		WM	−0.05141	0.01483	0.0005289						
		IVW	−0.08051	0.01677	1.57 × 10^−6^	IVW	2363.3	1.9 × 10^−210^			
		RAPS	−0.04956	0.02853	0.0829						
	HDL	MR Egger	0.05491	0.03144	0.08124	MR-Egger	1723.9	5.7 × 10^−112^	0.0003	0.0007	0.647
		WM	0.06454	0.01457	9.42 ×10^−6^						
		IVW	0.06788	0.01361	6.15 ×10^−7^	IVW	1724.5	8.02 × 10^−112^			
		RAPS	0.07245	0.03312	0.02911						
**Handgrip strength**	LDL	MR Egger	0.1764	0.1397	0.2085	MR-Egger	260.9	8.1 × 10^−07^	−0.0012	0.0017	0.463
		WM	0.06296	0.04887	0.1976						
		IVW	0.07787	0.03945	0.04839	IVW	261.8	8.7 × 10^−07^			
		RAPS	0.1377	0.1824	0.4515						
	VLDL	MR Egger	−0.4159	0.1625	0.01142	MR-Egger	388.3	5.1 × 10^−21^	0.0037	0.0019	0.061
		WM	−0.1884	0.04841	9.96 ×10^−5^						
		IVW	−0.1219	0.04634	0.008504	IVW	396.9	6.1 × 10^−22^			
		RAPS	−0.2736	0.1171	0.02068						
	HDL	MR Egger	0.4328	0.1841	0.01997	MR-Egger	551.8	1.6 × 10e^−44^	−0.0039	0.0022	0.079
		WM	0.03131	0.04646	0.5003						
		IVW	0.1211	0.05242	0.0209	IVW	562.5	6.2 × 10^−46^			
		RAPS	−0.03558	0.1262	0.7783						

Beta, beta-coefficients; HDL, high-density lipoprotein; IVW, inverse variance weighted; LDL, low-density lipoprotein; MR, Mendelian randomization; *Q*, Cochran’s *Q* statistic; RAPS, robust adjusted profile score; SE, standard error; VLDL, very low-density lipoprotein; WM, weighted median.

**Figure 3:**
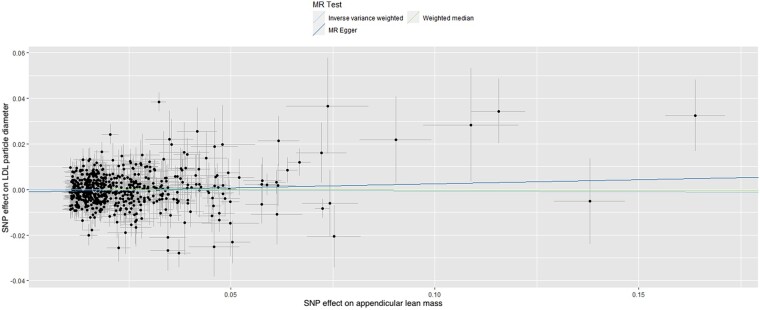
Scatter plot of the association of the effect of single nucleotide polymorphism-determined serum appendicular lean mass on low-density lipoprotein particle diameter. Each point represents an single nucleotide polymorphism, plotted by the estimate of single nucleotide polymorphism on appendicular lean mass (*x*-axis, kg) and the estimate of single nucleotide polymorphism on low-density lipoprotein particle diameter (*y*-axis, nm). The slopes of each line represent the potential causal associations for each method. LDL, low-density lipoprotein; MR, high-density lipoprotein; SNP, single nucleotide polymorphism.

These data suggest that each unit (kg) increase in ALM is associated with an increase of 0.07 nm in HDL particle diameter and a decrease of 0.08 nm in VLDL particle diameter. No statistically significant effect of ALM was observed for LDL particle diameter (MR-Egger: *β* = 0.035, SE = 0.03, *P* = 0.178, and IVW: *β* = −0.006, SE = 0.011, *P* = 0.575; *Table 1* and *Figure 3*).

**Figure 4:**
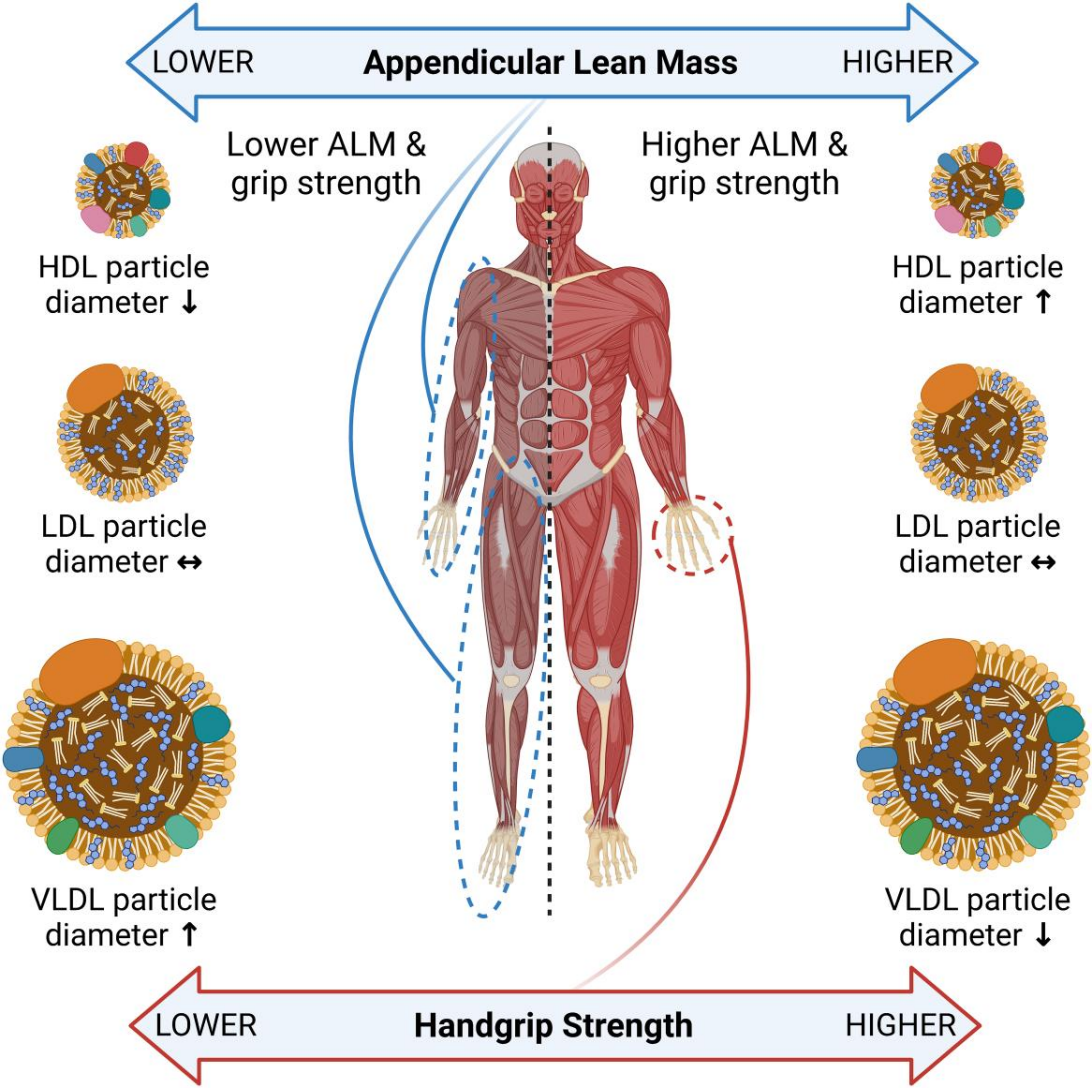
Graphical summary of results. As both appendicular lean mass and handgrip strength increase, HDL particle diameter increases while VLDL particle diameter decreases. No effect is seen on LDL particle diameter.

The MR analysis of HGS, displayed as beta-coefficient for interested outcomes per unit increase in HGS, showed a statistically significant positive effect on HDL particle diameter (MR-Egger: *β* = 0.433, SE = 0.184, *P* = 0.019, and IVW: *β* = 0.121, SE = 0.052, *P* = 0.021; *Table 1*) and a statistically significant negative effect on VLDL particle diameter (MR-Egger: *β* = −0.416, SE = 0.163, *P* = 0.011, and IVW: *β* = −0.122, SE = 0.046, *P* = 0.009; *Table 1*). These data suggest that each unit (kg) increase in HGS is associated with an increase of 0.12 nm in HDL particle diameter and a decrease of 0.12 in VLDL particle diameter. No statistically significant effect of HGS was observed for LDL particle diameter (MR-Egger: *β* = 0.176, SE = 0.139, *P* = 0.209, and IVW: *β* = 0.078, SE = 0.039, *P* = 0.048; *Table 1*). A graphical summary of the results can be seen in *Figure 4*.

The horizontal pleiotropy test, with very negligible Egger regression intercept, also showed a low likelihood of pleiotropy for all our estimations (all *P* > 0.05, *Table 1*), indicating a low risk of SNPs affecting multiple phenotypes via independent biological pathways. Furthermore, the result of the MR-RAPS was identical with the IVW prediction, which again indicated a statistically low chance of pleiotropy. Heterogeneity tests highlighted no trace of heterogeneity (*Table 1*). Furthermore, MR-PRESSO analysis did not indicate any outliers for any estimates. Results of leave-one-out method demonstrated that the links are not driven by any single SNP.

## Discussion

To our knowledge, this is the first study to reveal a potentially causal link between both genetically determined ALM and HGS with increased HDL particle diameter and decreased VLDL diameter.

Due to the relative novelty of the relationship of lipid particle diameter with muscle mass and strength, especially in terms of CVD risk, we cannot compare our results directly with the results of other studies. However, there are a number of observational studies and randomized controlled trials that have shown relationships between HDL-C and VLDL-C concentrations and either muscle mass or strength. For example, in a population of Japanese men and women (*n* = 991, age range 35–77 years), greater muscle thickness in the abdomen and thigh (quadriceps and hamstring muscles), relative to BMI, was significantly and positively associated with HDL-C concentrations in both sexes.^10^ Comparing a group of healthy men (*n* = 72, mean age 41 years) and men with CHD (*n* = 20, mean age 48 years), Tikkanen *et al.*^42^ observed that a greater percentage of slow twitch muscle fibres was associated with higher concentrations of HDL-C. In a further cross-sectional study, Wu *et al.*^43^ assessed the HGS of 17 703 Chinese men and women aged 40 years and older (median 45.2, interquartile range (IQR) = 51.3–59.2) and determined that reduced HGS was associated with reduced HDL-C, as well as other components of metabolic syndrome, including elevated triglycerides, blood pressure and fasting glucose levels.

Intervention trials have also revealed a relationship between increases in muscle mass and improved HDL-C levels. Ullrich *et al.*^44^ enrolled 25 young men (18–35 years) in an 8-week resistance exercise (RE) programme and reported that while body weight did not change significantly, muscle mass was observed to increase and was accompanied by a 14% increase in HDL-C concentrations [38.8–44.1 mg/dL (1–1.14 mmol/L), *P* < 0.001]. The increase in muscle occurred with a simultaneous decrease of body fat percentage, from 14% to 12.7%, which may independently affect HDL-C levels.^44^ Similarly, acute bouts of RE, known to elicit increases in muscle size and strength,^45^ have also been shown to reduce plasma VLDL triglyceride levels.^46^ However, to our knowledge, there are no interventions assessing the effects of RE on HDL or VLDL particle size.

Clinically, CVD risk is associated inversely with plasma concentrations of HDL-C, and positively with those of VLDL-C.^47,48^ Very LDL is an apolipoprotein B (apoB)-containing lipoprotein, which, along with LDL and intermediate-density lipoprotein, plays a significant role in the development of atherogenic plaques.^18,49^ The diameter of these apoB-containing lipoproteins is small enough for them to pass freely into the endothelial intima of blood vessels where, in the presence of endothelial damage or dysfunction, they may be taken up by macrophages.^49^ This leads to further inflammation and endothelial smooth muscle cell proliferation and the development of atherosclerotic plaques typical of CHD.^49^ In contrast to this direct effect, a larger VLDL diameter is associated with greater CVD risk,^19,20^ potentially via modification to other lipoproteins. Large VLDL particles, rich in triglycerides, may potentially play a role in the development of CHD through mechanisms such as increased formation of highly atherogenic sdLDL^50^ and increased catabolism of HDL.^51,52^ Indeed, the reduction of elevated triglyceride levels via agents such as icosapent ethyl has demonstrated efficacy in reducing the risk of cardiovascular events, although this effect may be related to reductions in total apoB-containing particles.^53^

Conversely, HDL is the key particle involved in reverse cholesterol transport, which transports excess cholesterol from peripheral body tissues to the liver for recycling or eventual excretion.^54^ It is via this mechanism, as well as through its antioxidant and anti-inflammatory actions, that HDL is thought to reduce the progression of atherosclerosis and the risk of CVD such as coronary artery disease (CAD).^54–56^ Furthermore, larger HDL particle size has been associated with a more favourable risk profile in the EPIC-Norfolk prospective population study.^22^ However, upon adjustment for other markers of CAD such as apoB and triglyceride levels, smaller HDL particle size was deemed to not contribute directly to CAD risk and may instead reflect a state of metabolic syndrome.^57^ In contrast, in a dietary study of extra virgin olive oil (EVOO) supplementation, older participants were found to have both lower cholesterol efflux capacity (CEC) and a predominance of smaller HDL particles compared with younger participants. After 12 weeks of supplementation with EVOO, the CEC of HDL was found to be improved through an increase in larger HDL and a decrease in smaller HDL particles, highlighting the role of particle size in HDL function.^58^ In contrast to HDL particle size, it should be noted, however, that while low serum HDL-C is frequently associated with poorer CVD outcomes,^59^ trials aimed at increasing HDL-C have consistently failed to show any clinical benefit in terms of CVD events.^60,61^ This has led to a revaluation of HDL’s mechanism of action with importance placed on CEC, rather than HDL-C concentration.^62^

Our study did not reveal an effect of increased ALM or HGS on LDL particle diameter. Due to LDL being the primary apoB-containing lipoprotein in circulation, it plays a major causal role in the development of atherosclerosis.^12^ Low-density lipoprotein particle size is known to contribute to CAD risk, with smaller particles having a longer plasma residence time, greater propensity to oxidation, and potentially infiltrating the endothelial intima more readily than larger particles and initiating an atherosclerotic cascade.^49,63^ Exercise training may lead to increases in LDL particle size^64,65^ by exerting effects on lipoprotein patterns through multiple mechanisms. Hence, this may not be directly comparable with our results that focus on the effects of muscle size and strength.

Both low skeletal muscle mass and low HGS are important risk factors for the development of CVD and indeed CVD- and all-cause mortality.^2–5^ More specifically, low skeletal muscle and low HGS may be independent risk factors for greater carotid intima-media thickness and high plaque score,^66^ highlighting their relevance in the development of atherosclerosis. However, the mechanisms by which muscle mass and strength may affect atherosclerosis are poorly understood. For example, muscle cells have been observed to efflux cholesterol to apoA1 during reverse cholesterol transport, which may contribute to elevations in circulating HDL-C,^67^ and greater muscle mass and strength are known to be associated with increased circulating HDL-C.^10,43^ Our results highlight a possible causal link between both greater ALM and HGS and increased HDL particle size, which may partially explain the mechanism by which muscle mass and strength contribute to reduced CVD risk.

Similarly, VLDL concentration is known to be acutely influenced by exercise and particularly RE,^46^ although, to our knowledge, no studies have associated muscle mass with VLDL-C concentration. However, the results of our study indicate that greater ALM and HGS are potentially causally associated with smaller VLDL particle size. On a per-particle basis, triglyceride-rich apoB-containing lipoproteins, such as large VLDL, may exert a greater risk of myocardial infarction than other apoB-containing lipoproteins,^68,69^ thus highlighting their relevance in CVD. Our results therefore suggest another potentially clinically significant benefit of increased muscle mass through reducing VLDL particle diameter and leading to a possible reduction in its atherogenic potential.

Exercise, in particular RE, is known to be the key driving force for increases in muscle mass and strength,^45^ and chronic exercise is associated with greater muscle mass and function in older adults.^70^ As such, it may be hypothesized that the deliberate use of exercise to improve muscle mass and function may lead to the changes in HDL and VLDL particle diameter that were determined in this study, conferring an improved risk profile for CVD. Further research is required to fully elucidate the effect of interventions to increase ALM and muscle strength on HDL and VLDL particle size and their relation to the risk of CVD. Furthermore, research is required to fully investigate the complex mechanisms that may link SNPs involved in muscle mass and strength with lipoprotein particle size and, potentially, clinical outcomes.

### Strengths and limitations

A major strength of our study was the large sample population study, with access to individual participant data of high validity from the UK Biobank cohort and with the relevant SNPs available for both ALM and HGS. The use of ALM instead of FFM is also of importance. Appendicular lean mass consists predominantly of skeletal muscle, while FFM is composed of skeletal, smooth and cardiac muscle, and bone and other non-fat tissues.^71^ Sarcopenia and the chronic conditions associated with it are defined by decreases in skeletal muscle mass and strength,^6^ and these deficiencies in muscle size and function can be ameliorated with appropriate exercise and nutrition interventions,^72^ highlighting the clinical relevance of ALM. The agreement of our results for the similar effects of both greater ALM and HGS on HDL and VLDL particle diameter further strengthens our findings. Additionally, the use of the MR approach allowed us to examine the potential causal effects of genetically determined ALM and HGS on lipoprotein particle size, largely without the disadvantages of confounding or reverse causation.

The use of segmental BIA for determining ALM in the UK Biobank cohort is a potential limitation of this study. Bioelectrical impedance analysis measurement accuracy is known to be affected by hydration status; however, the UK Biobank protocol did not specify any procedures to standardize hydration status before assessment. There exists the potential for such variation in hydration status to lead to inaccuracies in the ALM values attained.^73^ Evidence suggests that BIA may be less accurate at high BMI levels, which may be relevant considering the range of BMI included in the UK Biobank cohort.^74^ Furthermore, we acknowledge the potential for Type I error, particularly given the complexity of genetic associations and efforts to mitigate such errors were applied. A risk of Type I error remains inherent in this analytical approach and should be considered when discerning causality.

## Conclusions

There was a potentially causal association of both greater ALM and HGS, with increasing HDL particle size and decreasing VLDL particle size. Specifically, each unit (kg) increase in ALM or HGS is associated with an increase of 0.07 or 0.12 nm in HDL particle diameter, respectively, and a decrease of 0.08 or 0.12 nm in VLDL particle diameter, respectively. This causal association may offer possibilities for interventions aimed at improving CVD risk profile.

## Data Availability

No new data were generated or analysed in support of this research.

## Lead author biography

Dr Richard Kirwan has a PhD in clinical nutrition from Liverpool John Moores University where he is a lecturer in exercise physiology at the Research Institute for Sport and Exercise Sciences (RISES). His research revolves around the role of muscle mass in cardiometabolic health and particularly its relationship with blood lipids. His investigations focus on how resistance exercise and high-protein, cardioprotective dietary patterns may improve muscle mass and strength, improve blood lipid profiles, and reduce the risk of cardiovascular disease.
